# Conceptual Metaphors for Designing Smart Environments: Device, Robot, and Friend

**DOI:** 10.3389/fpsyg.2020.00198

**Published:** 2020-03-17

**Authors:** Jingoog Kim, Mary Lou Maher

**Affiliations:** Department of Software and Information Systems, The University of North Carolina at Charlotte, Charlotte, NC, United States

**Keywords:** metaphor, embodied interaction, human-computer interaction, interaction design, smart environments

## Abstract

A metaphor is a design tool that can support designers in forming and exploring new design concepts during the process of designing. Digital technologies embedded in built environments provide an opportunity for environments to be more intelligent and interactive. However, most architectural concepts associated with smart environments such as smart homes and intelligent buildings tend to focus on how advances in technology can improve the quality of the residential environment using automation and not on how people interact with the environment. We posit that conceptual metaphors of device, robot, and friend can open up new design spaces for the interaction design of smart environments. We present three metaphorical concepts that can frame new ways of designing a smart environment that focuses on interaction rather than building automation, each of which have distinct HCI techniques.

## Introduction

New technologies allow our built environment to become intelligent and interactive. Embedding computation in physical environments changes our environment from a static to an interactive space. There are many concepts related to emerging new technologies in architecture such as building automation, smart homes, adaptive buildings, intelligent buildings, and interactive architecture. However, there are few studies about a theoretical and methodological framework to understand and expand the design space of interactive designs in a built environment. Therefore, designing smart environments requires a new foundation to guide in the conceptualization of novel designs using concepts that emerge from human–computer interaction (HCI). Using metaphors is a design technique that can frame new design spaces for interactive designs and support designers in creating novel interaction experiences. Metaphorical references can also assist users in perceiving affordances of novel designs. We look to metaphorical design to provide a common mental model for both designers and users in the transition from traditional to smart environments.

Interaction between an occupant and an interactive system in a built environment relies on embodied interaction, an approach to HCI designs emphasizing everyday experience as a foundational concept for HCI. As computers are increasingly embedded in physical environments, embodied interaction has expanded to ubiquitous computing with the development of new technologies. Understanding embodied interactions that can be adapted to smart environments thus is essential in order to identify new design spaces for smart environments. We claim that characterizing embodied interaction with the conceptual metaphors can provide a basis for designing smart environments.

In this paper, we present three conceptual metaphors as a basis for characterizing smart environments: device, robot, and friend. We provide a review of existing embodied interaction designs that can be adapted to smart environments from the perspective of these three metaphorical concepts to expand on the ways in which each metaphor is distinct and enables the creation of new designs that provide a consistent mental model for designers and users. We discuss how each conceptual metaphor frames new designs for smart environments through educational experiences. Finally, we show how framing a specific design with each of the conceptual metaphors leads to different interactive experiences.

## Metaphors in HCI

Metaphors are widely used in HCI as a vehicle for representing and developing designs. A metaphor is a mapping process from a familiar object to an unfamiliar object, and it provides the framework to familiarize an unknown concept through a mapping process. The role of a metaphor in HCI is to facilitate developing, maintaining, and learning the conceptual foundation of the interactive design as well as orienting the user with it (Saffer, 2005). Using metaphors involves the exploration and expression of an idea that is integral to design generation and innovation (Brady, 2003). In this perspective, metaphors can be used as a tool in the design process to understand new topic areas or as a means to create new ideas about familiar subjects. They enhance our perception by transforming our sense of reality (Ricoeur, 1991), and new metaphors can create a comprehensive conceptual system (Lakoff, 1993). Metaphors can also assist in engaging the designers’ existing mental models. A mental model is an organized collection of data that acts as a representation of a thought process (Marcus, 1992, 1995). Mental models refer to analogs of real-world processes, including some other aspects of real-world processes (Gentner and Gentner, 2014). Analogical reasoning, an inference method in design cognition (Gero and Maher, 1991), is a method for developing designs that can lead to unexpected discoveries. In conceptualizing a new interactive system, a metaphor can be a useful tool for establishing a common mental model for designers. We claim that the positive impact of metaphors in HCI can be beneficial in conceptualizing smart environment. In this paper, we describe how metaphorical design enables the conceptualizing of a smart environment design in which different metaphors lead to new conceptual spaces.

The most well-known metaphor in HCI is the desktop metaphor, which represents the user interface in a way that is similar to interactions concerning certain objects, tasks, and behaviors encountered in physical office environments. Despite the desktop metaphor still being predominant in the personal computing environment, it shows problems and limitations (Moran and Zhai, 2007; Antle et al., 2009; Houben, 2011; Jung et al., 2017) in being adapted into recent interaction designs (e.g. tangible interaction, speech interaction, and mixed reality) since it focuses on the personal computing and visual interface design. While much of the research done on metaphors in user interface design has been focused upon the use of metaphors in the design of visual communication elements of the graphical user interface (GUI) and in understanding users’ mental models of such interfaces (Voida et al., 2008; Antle et al., 2009; Houben, 2011), some researchers have pointed out the limitations of the desktop metaphor and proposed alternative metaphors (Abowd and Mynatt, 2000; Moran and Zhai, 2007; Antle et al., 2009; Jung et al., 2017). They showed several dimensions (e.g. context, modality, materiality, and affordance) of alternative metaphors as a systematic strategy to emphasize a new interactive form which can be conceptualized with metaphorical mapping. A smart environment provides potential design spaces that are yet to be fully explored and understood. We posit that new forms of smart environment can be characterized by comprehensive metaphors that can uncover potential design spaces for a smart environment by providing a common mental model.

## Smart Environments and Embodied Interaction

Smart environments are associated with recent architectural concepts based on new technologies. From the HCI perspective, a smart environment is based on embodied interaction involving physical movements of occupants and spatial aspects of the environment. In this section, we describe architectural concepts associated with smart environments and embodied interaction that can be adapted to smart environments.

### Smart Environments

Smart environments reflect recent architectural phenomena that embed computation in built environments, providing dynamic spaces to support a range of humanistic functions. There are many architectural concepts associated with smart environments: intelligent buildings, building automation, sentient buildings, smart homes, responsive architecture, adaptive buildings, kinetic architecture, and interactive buildings. These architectural concepts reveal different perspectives of smart environments such as purposes, functions, building components, and interactivity.

The concepts of intelligent buildings, building automation, and sentient buildings are associated with automation in a built environment. An intelligent building is a building using integrated sensor systems that maintain optimum performance by automatically responding and adapting to the operational environment and user requirements (Callaghan, 2013). Building automation is a system for monitoring and controlling mechanical, security, fire and flood safety, lighting, and HVAC systems in a building (Achten, 2014). A sentient building is a sensor-driven monitoring and controlling system that has an internal representation of the building from which it derives control strategies (Mahdavi, 2004; Achten, 2014). These concepts mostly focus on automation for environmental comfort, safety, security, privacy, energy use, and efficiency. The smart home is a part of building automation that applies to residential buildings (Achten, 2014). Although the smart home is a similar concept to building automation, it provides more opportunities for occupants to actively control their environment through handheld devices connected with the home environment. In other words, smart homes involve not only automation but also direct interaction to support ease of control in the living environment and occupants’ routine activities. Responsive architecture, adaptive buildings, and kinetic architecture are associated with environmental comfort and sustainability. These concepts typically refer to smart facade systems that automatically react to internal and external environmental conditions with physical movements of building elements (Achten, 2014; Fortmeyer and Linn, 2014).

The architectural concepts associated with smart environments provide opportunities to improve the quality of the residential environment including environmental comfort, ease of control for living, security, better building performance, and sustainability. However, most concepts focus on automation rather than how people interact with the built environment. We look to conceptual metaphors and metaphorical design that can provide a new approach to rethinking the smart environment for novel interactive experience designs.

### Embodied Interaction

Interaction between a user and a system in a built environment is strongly associated with embodied interaction since it depends on the user’s physical body movements and spatial aspects of the environment. Embodied interaction is interaction between computer systems and people that involves our physical bodies for the interaction in a natural way, for example, gestures. Dourish (2001) described that embodied interaction is about “how we understand the world, ourselves, and interaction comes from our location in a physical and social world of embodied factors.” Embodiment leverages users’ body movements to facilitate interaction with a computational system embedded within a space or physical object which involves tangible computing, social computing, mixed reality, responsive environments, pervasive computing, and physical computing (Antle et al., 2009). Embodied interaction emphasizes a way to combine perspectives of tangible interaction and social computing (Dourish, 2001). Recent embodied interaction designs expand to new technologies such as wearable computing, kinesthetic sensations, full-body interaction, multimodal interaction, and conversational embodied agents (Hobye and Löwgren, 2011).

Embodied interaction is also associated with metaphorical design. Lakoff and Johnson (2008) argue that abstract concepts rely on metaphorical extensions of embodied schemata, which are mental representations of recurring dynamic patterns of bodily interactions that structure the way we understand the world. Embodied schemata based on recurring patterns of bodily experience facilitate reasoning about abstract concepts in metaphorical thinking. An embodied metaphor is a mapping process between a source domain of embodied experiences and a target domain of an abstract concept. Therefore, an embodied metaphor extends embodied schemata to structure and understand abstract concepts (Antle et al., 2009; Bakker et al., 2012).

Rompay et al. (Van Rompay and Hekkert, 2001; Van Rompay et al., 2005) investigated the effect of embodied schemas in product design with several experiments based on the theory of metaphor developed by Lakoff and Johnson (1999, 2008). Rompay et al. examined the relationship between embodied schemas (e.g. inside–outside), the product expressions (e.g. secure–insecure), and design properties (e.g. size, material) through experiments that ask subjects to rate product expressions associated with embodied schemas and/or design properties of various designs (e.g. different shapes of chairs). The results showed that product expressions are associated with the same underlying embodied schemas in the spatial and material features of products. That means bodily experiences can structure our understanding of products and product characteristics can be derived from schemata that affect a product’s expression in a consistent manner. In this perspective, embodied schemas, a basis for embodied metaphors, can provide guiding principles for designing physical products, and designers can use embodied metaphors as a design tool.

While Rompay et al. focused on how the properties of product design can be associated with embodied schemas, there are several studies that investigated the effect of embodied metaphors in interaction designs. Antle et al. (2008, 2009) presented an interactive musical sound-making environment, Sound Maker, for learning about abstract sound concepts. They used the ontological metaphor “music is body movement” to map bodily movement to the sound parameters of volume, tempo, and pitch. From the comparative study of the same interactive system implemented with and without an embodied metaphor, they provided evidence that an embodied metaphor supports the creation of systematic relationships between specific user actions and specific system actions.

Bakker et al. (2012) have presented a tangible system, MoSo Tangibles, for learning about abstract sound concepts (pitch, volume, and tempo). This is a similar approach to that of Sound Maker in terms of using embodied metaphors for mapping bodily movements to sound parameters. MoSo Tangibles uses multiple tangible artifacts and embodied schema for hand movements, while Sound Maker uses a single artifact for full-body interaction. This case was revealed to elicit a set of embodied metaphors that children may use in their reasoning about abstract concepts related to sound parameters.

Abrahamson and Trninic (2011) used embodied interaction based on spatial metaphors of bodily experience for mathematical learning. The interactive display called Mathematical Imagery Trainer measures the height of the user’s hands to give visual feedback for rational-number problem solving. The results of the study showed that spatial embodied metaphors facilitate mathematical problem solving.

Hemmert and Joost (2016) used embodied metaphors for interactions with mnemonic objects in live presentations. They focused on the use of container metaphors for bimanual and spatial interactions in the context of live presentations (i.e. topics are picked up, and one goes through a series of points and comes to a conclusion). The user can activate a topic by picking up the corresponding object. Each topic consists of multiple points, and the user can activate a point by walking up to its position on stage. This case reveals the use of embodied metaphors for a multimodal interaction.

Löffler et al. (2016) applied conceptual metaphors to the realm of colors for tangible interaction design (e.g. Important is a dark color). They investigated color-to-abstract mappings to tangible interaction design: whether colors can substitute haptic object characteristics (i.e. the size, weight, or temperature of tangibles) when conveying abstract meaning. The results suggested that color can replace haptic attributes in metaphoric mappings.

The studies on embodied interaction and embodied metaphor show the potential and effect of embodiment on designing physical products. The insights from the studies indicate that embodied metaphors can provide guiding principles for physical products and can extend to smart environments. However, the studies on embodied schemas focus on physical design properties rather than interactivity, and the studies on embodied metaphors for interactivity tend to use metaphors for mapping body movements while focusing on a specific tangible and gesture interactions. We build on this foundation to introduce metaphorical concepts that can guide the designer to explore the design space of embodied interaction technologies to enable a common mental model for the designer and the user in a smart environment.

## Conceptual Metaphors and HCI Framework for Characterizing Smart Environments

We present three metaphorical concepts as a basis for characterizing smart environments through analogical reasoning: device, robot, and friend (Kim et al., 2018). Interaction between a user and a computer as a phenomenon can be a basis for a new metaphor in the sense that metaphors in HCI provide familiar mental models of how the computer can be used. In order to identify new metaphors for smart environments, we focused on the role of the user and the role of the system when performing a task. We categorized three different perspectives of embodied interaction for the potential conceptual metaphors: performing tasks by direct control, performing tasks by automation, and performing tasks by assistance. Direct control for performing a task represents the fact that users actively control an activity, environment, and information with an embodied interaction (e.g. touch, gesture, and tangible). In this case, the user initiates and leads the interactions between the user and the system. We established the device metaphor for direct control to emphasize the characteristics by which a user performs a task using an interactive design like a device. The performing task by automation represents that an interactive system actively performs tasks detecting external conditions and analyzing data without human control. In this case, the interactive system initiates the interaction between the user and the system. We use the robot metaphor to highlight the characteristics of automation and autonomous features. The performing task by assistance represents that an interactive system actively intervenes in certain activities done by the users (e.g. personal assistance using AI technologies). In this case, both the user and the system can initiate and lead the interactions between them. We use the friend metaphor for the performing task by assistance for emphasizing the characteristic of a human-like manner for natural interaction.

A conceptual metaphor for HCI is associated with interaction styles and modalities (Satzinger et al., 2011). For example, the desktop metaphor reflects direct manipulation where users interact with objects on display screen menus and WIMP interfaces. The document metaphor involves browsing and entering data in electronic documents such as browsing, WWW, hypertext, hypermedia, forms, and spreadsheets. The dialog metaphor carries on a conversation with speech or natural language. In order to characterize a smart environment with the presented metaphors, we adapted the concepts of interaction type, interface type, and affordance as a framework for representing HCI techniques.

• Interaction type: the ways by which a person interacts with a product or application (i.e. instructing, conversing, manipulating, exploring, and sensing) (Rogers et al., 2011; Kim et al., 2018).
• Interface type: technologies that enable and support the interaction (e.g. WIMP, GUI, touch, speech, wearable, tangible, AR, and VR) (Rogers et al., 2011).
• Affordance: the action possibilities of a user when the user interacts with a designed artifact (e.g. pressing a button or turning a knob) (Norman, 1988; Rogers et al., 2011).

There are numerous embodied interaction designs that use different modalities and interaction techniques. As recent embodied interaction expands to new technologies such as wearable computing, kinesthetic sensations, full-body interaction, and conversational embodied agents (Hobye and Löwgren, 2011), many of the recent embodied interaction designs tend to use multimodal interaction.

## Characterizing Smart Environments With Conceptual Metaphors

**FIGURE 1:**
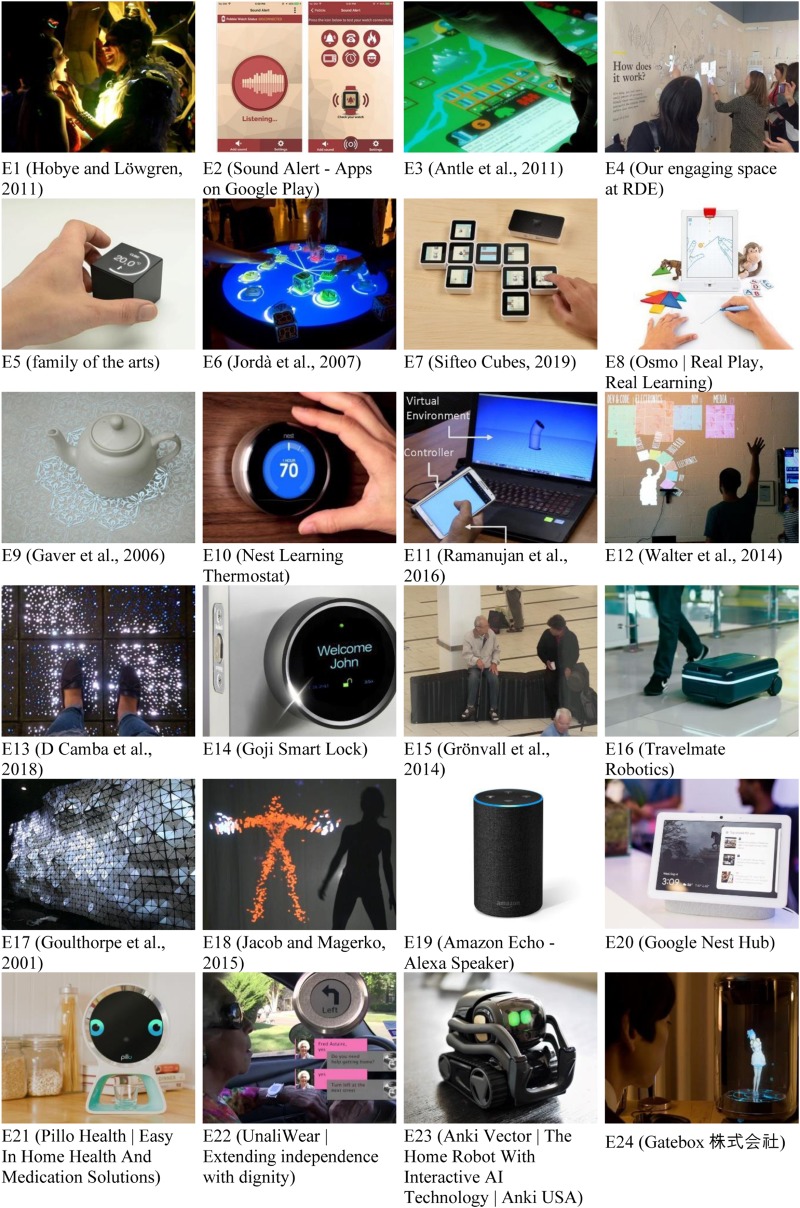
Images of 24 existing designs.

As a basis for characterizing smart environments, we review a range of existing embodied interaction designs through the perspectives of HCI techniques (i.e. interaction type, interface type, and affordance) and our three conceptual metaphors. Our selection of designs is based on a sample of the literature using a search for research publications in Google Scholar. Our search terms include multiple ways of describing embodied interaction (e.g. “embodied interaction,” “tangible interaction,” “embodied metaphor,” “multimodal interaction,” “mid-air gesture,” “haptic,” “public display,” “full-body interaction,” “embodied AI,” etc.). We also sampled interaction designs by searching for websites and commercial products using terms such as “embodied interaction,” “tangible interaction,” “personal assistant,” and “multimodal interaction” to include in our review of implemented embodied interaction designs. In this review, we included examples of existing embodied interaction designs categorized into embodied types by decreasing the scale of physical involvement in the users’ interactions: full body (e.g. mid-air gesture, interactive wall), tangible using physical objects [e.g. tangible user interface (TUI), organic user interface (OUI)], kinesthetic (e.g. haptic, touch), and conversational (e.g. conversational embodied agent, AI agent). There were no specific exclusion criteria for the results of articles and commercial products, but we avoided including multiple similar designs in a single category. In this review, we examine 24 existing designs (Figure 1) for alignment with three metaphorical concepts, embodied types, and interaction types.

**FIGURE 2:**
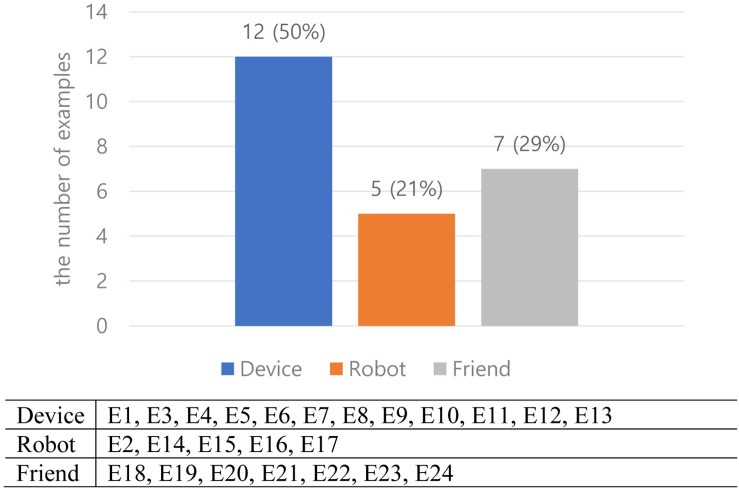
Embodied designs categorized by metaphors.

Figure 2 shows the percentages of designs categorized by our three metaphorical concepts: 50% of device (12 designs), 21% of robot (5 designs), and 29% of friend (7 designs). Most designs in the device metaphor are wearable and have multi-touch displays, a TUI, an OUI, and mid-air gesture-based public display. Most designs in the robot metaphor use everyday objects (e.g. smart lock, shape-changing bench, shape-changing wall, and robotic suitcase). Most designs in the friend metaphor are different types of personal AI assistants using speech, screen, hologram, and physical robot types of interfaces.

**FIGURE 3:**
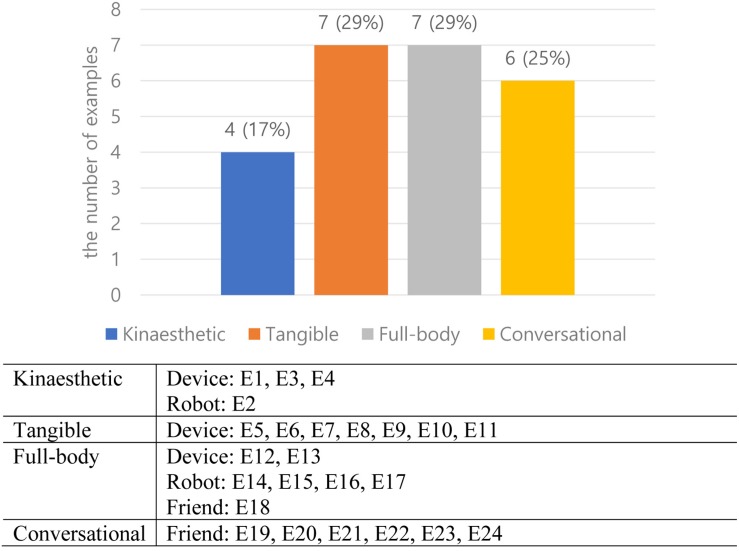
Embodied designs categorized by embodied types.

Figure 3 shows the percentages of designs categorized by embodied types: 29% of full body (two designs of device, four designs of robot, one design of friend), 29% of tangible (seven designs of device), 17% of kinesthetic (three designs of device, one design of robot), and 25% of conversational (six designs of friend). The results indicate that kinesthetic and tangible types are mostly associated with the device metaphor, the full-body type is related to the robot metaphor, and the conversational type is associated with the friend metaphor.

**FIGURE 4:**
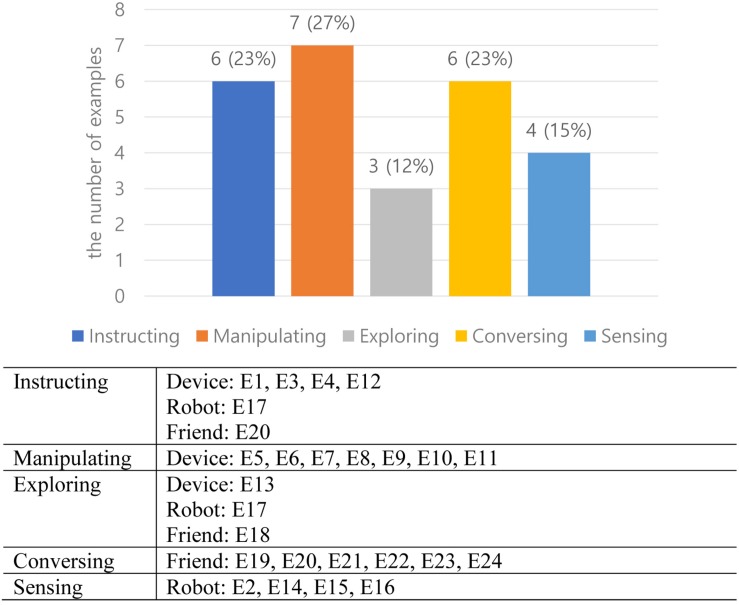
Embodied designs categorized by interaction types.

Figure 4 shows the percentages of existing designs categorized by interaction types: 23% of instructing (four designs of device, one design of robot, one design of friend), 23% of conversing (six designs of friend), 27% of manipulating (seven designs of device), 12% of exploring (one design of device, one design of robot, one design of friend), and 15% of sensing (four designs of robot). Two out of 24 designs use two interaction types, and thus, the total number of interaction types in the chart is 26. Most embodied interaction such as TUI, touch, and mid-air gesture rely on instructing and manipulating types of interaction. However, the analysis shows that exploring, sensing, and conversing types of interaction are not a small portion of embodied interaction. The embodied interaction designs that use those types of interaction reflect recent emerging technologies that use multimodal interaction. The analysis also indicates that instructing and manipulating types are mostly related to the device metaphor, the sensing type of interaction is related to the robot metaphor, and the conversing type of interaction is strongly associated with the friend metaphor. Exploring types of interaction can be applied for all three metaphorical concepts.

**FIGURE 5:**
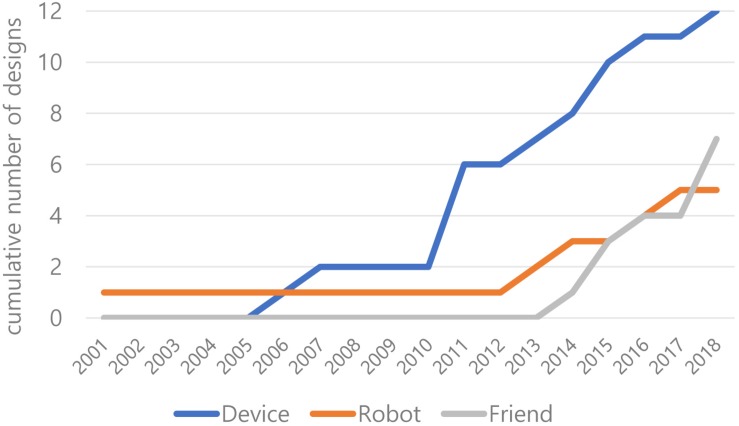
Emergence of three categories of design.

Figure 5 shows the emergence of existing designs categorized by three metaphorical concepts. While the designs based on the device metaphor gradually increase from 2005, the designs based on the robot and friend metaphors mostly appear from 2012 and 2013. The designs based on the robot and friend metaphors tend to combine emerging technologies with embodied interaction using sensing and conversing types of interaction with a multimodal interface type. From this perspective, we assume that the robot and friend metaphors can be beneficial in providing a common mental model and uncovering new designs spaces for future embodied interaction designs.

Table 1 shows the mapping of interaction types, interface types, and affordances onto the metaphorical concepts, as realized in the 24 embodied interaction designs. In terms of interaction style and modality, the device metaphor is strongly associated with direct manipulation, the robot metaphor involves automated features, and the friend metaphor reflects conversational interaction and a human-like manner. The distinct characteristics of each of these metaphors lead to different types of interactions, interfaces, and affordances.

**TABLE 1:** Metaphorical concepts characterized by interaction type, interface type, and affordance.

	**Performing task by direct control: device**	**Performing task by automation: robot**	**Performing task by assistance: friend**
Interaction type	Instructing, manipulating, exploring	Exploring, sensing	Conversing, exploring
Interface type	Tangible user interface (TUI), mid-air gesture, wearable, organic user interface (OUI)	Robot, appliance, ambient device, OUI w/automation	Speech, appliance w/dialog, wearable w/dialog, multimodal
Affordance signifier	Digital elements of interface (e.g. icons and graphics for touch), physical shape of interface (e.g. a cube for flipping)	Physical movement of interface (e.g. automatic unlocked door knob for opening the door), automated information changes (e.g. automatic activated screen for face ID)	Speaking information and instruction (e.g. speaking “What can I help you with?”), emotional speech, tone, pace, pitch of speech, actions/gestures of virtual assistant (e.g. beckoning, dancing)

This analysis of existing designs has identified how each metaphor is associated with specific interaction types, interface types, and affordances that can be a guide to future embodied interaction designs. In this section, we describe examples of existing embodied interaction designs associated with each metaphor and how each metaphor structures a smart environment with embodied interaction by interaction types, interface types, and affordances.

### Smart Environment as a Device

A smart environment with embodied interaction is a device on a much larger scale, extending the concept of a smartphone or smart appliance to an interactive design. This metaphor represents performing tasks through users’ direct control. It encompasses providing better service, performance, and ease of control by using an interactive design as a device. This metaphor emphasizes multiple purposes and operations in a single device. Thus, it provides insight into how users can control information, activity, and environment with an interaction. The device metaphor represents direct manipulation which involves instructing, manipulating, and exploring types of interaction. It encompasses the interface types TUI, mid-air gestures, and OUI. The interfaces for this metaphor use GUI elements and physical shapes of the interface for the affordance and the signifier.

**FIGURE 6:**
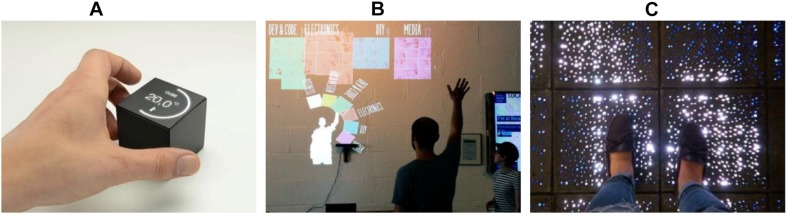
Embodied interaction designs in device metaphor. **(A)** Tangible controller (family of the arts), **(B)** interactive public display (Walter et al., 2014), **(C)** interactive floor display (Camba et al., 2018).

Figure 6 shows examples of the device metaphor for smart environments. Cube (family of the arts), shown in Figure 6A, is a tangible home controller that users can rotate, shake, spin, tap, and swipe to control different parts of the home such as lighting, heating, and cooling. This is an example of the device metaphor being applied to control an environment using tangible interaction, and Cube looks like an actual device. Cube uses the manipulating type of interaction through physical actions such as rotating, shaking, and tapping the cube. The interface type of Cube is a combination of GUI, TUI, and appliance. Cube uses a GUI similar to existing mobile apps such as a clock screen with a battery icon, a music icon, and a dial graphic. This familiar GUI helps users to understand the usage of Cube. The type of technique is a tangible interaction to manipulate Cube using physical actions. In this case, the tangible interaction is used to control multi-functions in a place, and it can be compared to controlling multi-functions by a smartphone and voice commands through a smart speaker (e.g. Amazon Echo)^1^. This type of platform is an appliance used as a smart home device. The affordances and signifiers are icons/graphics (digital) and the surfaces/shape of Cube (physical). For example, a surface of Cube shows a dial type of controller graphic, and the dial makes the user rotate Cube (e.g. turning up the volume). Another example is that each surface has different icons for different functions (e.g. blind, light), and it makes the user spin Cube to select other functions.

Figure 6B is an interactive public display that uses mid-air gesture interaction (Walter et al., 2014). A public display and the use of mid-air gestures are analogous to a mobile device in the sense that they provide experiences similar to mobile interaction such as with smartphones and tablet PCs. Touch interaction on mobile devices can be transferred to mid-air gesture interaction in a similar way (e.g. tap of touch to dwelling of gesture), and the use of GUI and presentation of information in mobile devices can be transferred to a public display in a similar way. The interactive public display using mid-air gestures uses instructing types of interaction (e.g. selection gesture and confirmation gesture). The interface types are mid-air gestures and a shareable display. The affordances and signifiers are mirror images of the user and the shape of items.

The last example is an interactive floor display (Camba et al., 2018), shown in Figure 6C. This example presents a larger scale of the device metaphor. The interactive concrete tiles with LEDs provide a natural visualization such as pedestrian navigation, advertising, and entertainment. In this example, the building as a device can use an entire floor of the building or multiple spaces as a user interface. The interaction between users and device is also expanded as walking through the information rather than touching/manipulating a device in a space, but is still analogical (e.g. touching/pressing button vs. walking tiles). The interaction floor display uses exploring types of interaction by allowing the users to walk on concrete tiles; users explore physical spaces on the floor to get information. The interface types are OUI, touch (foot), and physical objects (LED concrete tiles). The interactive floor uses various graphics such as figures, text, and logos. The graphics are presented when the walking action is provided as input. In this case, the affordances are guiding and walking, and the affordance signifiers are graphics and the positions of the highlighted tiles. For example, presenting arrow graphics for navigation purposes induces users to follow the arrows. Some highlighted tiles that are a bit further away from a user make the user look at the tiles or walk through the tiles.

In the device metaphor, the examples use instructing, manipulating, and exploring interaction types. A significant characteristic of the device metaphor is manipulatable/direct interaction, and this characteristic is associated with instructing, manipulating, and exploring types of interaction. For the interface type, the examples use some generalized GUI or graphics to help users’ understanding even if they use different techniques and platforms. The examples show a combination of digital affordances and physical affordances. The digital affordances tend to be generalized affordances such as icons, buttons, and graphics to take advantage of familiar affordances. On the other hand, the physical affordances are unique and are associated with the size and shape of the physical platform. The interactive floor is a good example for a combination of physical affordances and digital affordances.

### Smart Environment as a Robot

A smart environment with embodied interaction is a robot in the sense that it is an intelligent machine capable of performing tasks without explicit human control. An interactive system based on this metaphor reacts to external changes autonomously to perform tasks. This metaphor emphasizes automation, autonomous decision making, artificial intelligence, and the physical actions of a robot. It reflects learning and adapting through interaction with users and sensors. The robot metaphor involves exploring and sensing interaction types for automated features that detect users and analyze data to take a system action. It includes the interface types of robot, appliance, ambient device, and OUI with automation. The affordances for this metaphor are physical movements of the interface (e.g. automatic unlocked doorknob for opening door) and automated information changes (e.g. automatic activated screen for face ID).

**FIGURE 7:**
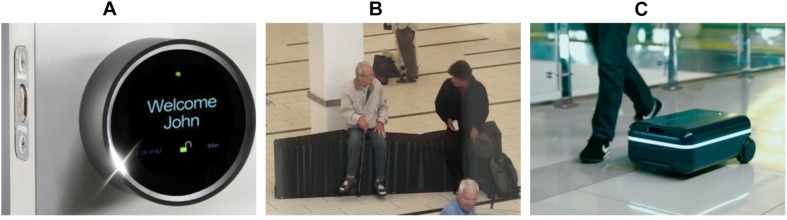
Embodied interaction designs in robot metaphor. **(A)** Smart door lock (Goji Smart Lock, 2014), **(B)** shape-changing bench (Grönvall et al., 2014), **(C)** robotic suitcase (Travelmate Robotics, 2018)^3^

Figure 7 shows examples of the robot metaphor with different scales and purposes in a smart environment based on embodied interaction. The smart door lock (Goji Smart Lock, 2014)^2^, as shown in Figure 7A, is a small-scale version of the robot metaphor using a physical object and represents automation and autonomous features of a smart home, automatically detecting occupants and opening the door. The building component, the smart door lock, is a robot used to manage home security in this case. The smart door lock uses the sensing type of interaction so that the door lock detects a user when the user comes close to the door. The interface types are physical object, GUI, and appliance. Although the door lock presents some text and icons (GUI), the interface mostly depends on sensors for the major function of security and the mechanical components as a robot. The digital affordance is the lock icon (open/close), and it allows the user to manually control the door lock. The door lock is a round type of knob, and it is used as a physical affordance to turn the knob.

A shape-changing bench (Grönvall et al., 2014) called coMotion is a public bench that is installed in public spaces, as shown in Figure 7B. The bench, as a robot, changes shape by itself by detecting people sitting on it and their body movements to encourage their social communication. The shape-changing bench uses two types of interaction: sensing and exploring. Basically, the interaction between the user and the bench is that of sensing. For instance, the bench detects a sitting user and then changes its shape. In another case, the bench changes its shape when the user changes his/her sitting position. However, if the user explores the shape of the bench to find a more comfortable position, the interaction type would be that of exploring in this case. The interface types are physical objects and shareable. The bench uses only a physical object with sensors and mechanical elements. Therefore, there are no visual interfaces and affordances. The physical affordance is the shape of the bench, such as the high and low positions of the seat. The shapes allow users to decide their sitting position and posture.

The robotic suitcase (Travelmate Robotics), Figure 7C, shows an everyday object that is mapped to a physical robot. As a mobile robot, the suitcase follows the user by detecting the user’s location. The robotic suitcase also reacts to the user’s gestures such as beckoning him/her to come closer and gesturing at him/her to turn around. The interaction type of the robotic suitcase is that of sensing by detecting the user’s location and body movement. The interface types are physical objects and mobile, which is an automatic system that consists of sensors and mechanical components. The affordance and signifier is the LED lighting that indicates the moving direction.

In the robot metaphor, the examples depend on the sensing type of interaction and the robot type of interface, since the major characteristic of the robot metaphor is a physical automation system for achieving certain functions. However, the interaction type can be that of exploring when the purpose/function of the design involves more user actions. The affordances in the robot metaphor are physical and depend on the embodied interactions used and their shapes.

### Smart Environment as a Friend

A smart environment with embodied interaction is a friend in the sense that the interaction centers around advising and supporting the users. Interactions based on this metaphor have the role of supporting activities within a specific context. The primary characteristic of this kind of interaction is an open-ended structure and outcome, which are characterized by meandering interaction. This metaphor represents performing tasks with assistance regarding the supporting users’ activities. This metaphor emphasizes conversation and the perspective of a friend being offered. It reflects research in personification, effect, and artificial intelligence as well as co-learning over time. The friend metaphor represents conversational interaction, which involves conversing and exploring types of interaction that reflect a human-like manner. It includes the interface types of speech and multimodal. Affordances and signifiers for this metaphor differ from traditional affordances and signifiers that mostly focus on the shape of a familiar object as a visual cue. Instead, this metaphor uses speaking manner (e.g. instructional speech, emotional speech, speech tone, speech pace, and speech pitch), facial expressions (e.g. angry face, crying face), and the actions of a virtual assistant (e.g. waving hello, dancing, and drinking coffee).

**FIGURE 8:**
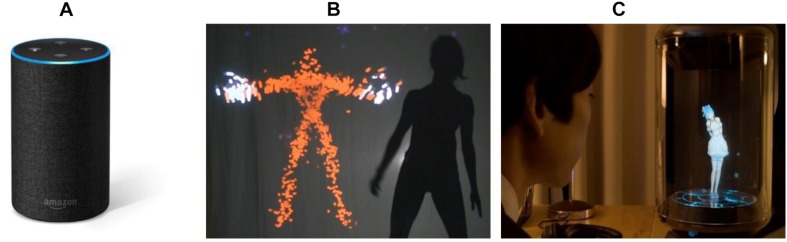
Embodied interaction designs in friend metaphor. **(A)** Smart speaker (Amazon Echo Alexa speaker), **(B)** AI agent for improvisation (Jacob and Magerko, 2015), **(C)** virtual home assistant robot (Gatebox, 2019 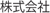)^4^.

There are many home assistants termed smart speakers nowadays with the increasing use of AI technologies, and they support users’ everyday activities like setting timers, providing news, and playing music in a human-like manner, such as using voice interaction, for instance, as depicted in Figure 8A. In this case, the AI agent is mapped to a friend that supports the user’s routine activities using natural language in a human-like manner, although most smart speakers use voice interaction in a human-like manner, where a human-like manner can involve other types of interaction such as virtual character, text (chatbot), image, etc. Amazon Echo (Amazon Echo Alexa speaker) is a smart home device, and the user interacts with the virtual assistant called Alexa through voice conversation, like for instance, “Alexa, what’s the weather report for tomorrow?” The interaction type of Amazon Echo is that of conversing, and the interface types are speech and appliance. In this case, affordances are the voice responses of Alexa such as communicating information and instructions by speaking.

The Viewpoints AI installation, Figure 8B, is a participatory interactive installation that allows humans to collaborate with a virtual agent to improvise movement-based performance pieces together in real time (Jacob and Magerko, 2015). The virtual agent analyzes participant movements and improvises responses based on past interactions with people. In this case, the AI agent is mapped to a friend to act as a dance partner and co-creative performer. The interaction type of this example is that of exploring, which explores the movements of a virtual agent, including physical and spatial movements. Although it uses the exploring type of interaction, the interaction is analogous with conversing in terms of communicating through dancing and co-creating performance. The interface types are mid-air gestures and shareable display. The affordances are bodily movements of the virtual agent (e.g. mimicking the user’s dance, changing the tempo of the dance, and suggesting a new dance based on the user’s movements).

Gatebox (Gatebox 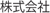), displayed in Figure 8C, is a virtual home assistant that uses a hologram and is an example of virtual character–type human-like manner. Unlike the first two examples, the virtual character of Gatebox uses a combination of speech interaction and whole-body actions like an actual person. In this case, the bodily actions of the virtual character can play an important role as an affordance, for instance, having teatime with the user. In addition, the virtual character can respond to and send a text message to the user. Thus, it affords more complicated users’ actions, emphasizing emotional relationships between users and the virtual assistant even if it provides functions similar to those of Amazon Echo. Gatebox uses a conversing type of interaction. However, it also uses a more emotional human-like manner based on the interface type, while Amazon Echo uses only speech interaction with functional purposes in a human-like manner. Gatebox uses mixed reality with a hologram as an embodied interface, and the technique used is a multimodal interface including speech and display. Gatebox involves many different affordances since it uses a visual and emotional assistant. The virtual assistant can gesture and speak emotionally to induce a user action. Some examples of these gestures are greeting gestures, pointing icons/information, running to the user, etc. The gestures make the user greet the assistant, look at the information, and take an action according to that information. Examples of emotional talking through speech and text message are “come home early” and “hmmm….” Talking makes the user respond or take an action.

In the case of the friend metaphor, the interaction type should be conversing since the essential characteristic of the friend metaphor is a natural dialog. In the examples given, speech is the common interface type for conversing interaction. However, gestures can be also considered for an interface type such as dancing. Another aspect of the interface type is visual elements such as the visual assistant of Gatebox. Although Gatebox uses mixed reality with a virtual character, it can be expanded to other types of interface such as wall/floor display and 3D projection. In addition, both of the examples are home device types of appliances. However, potential designs can be embedded into building components such as walls, windows, and doors. Lastly, the use of visual assistants shows potential affordances that involve the emotional aspect of a human-like manner. Further research is needed for identifying potential affordances.

## Framing New Designs of Smart Environments Using Conceptual Metaphors

### Educational Scenarios

In order to explore the effect of the conceptual metaphors in design scenarios, we used the conceptual metaphors in educational settings. We focused on two perspectives of conceptual metaphors: how people adopt the metaphors as a mental model from the user’s perspective and how designers apply the three metaphors to develop their conceptual design from the designer’s perspective. In this section, we describe the factors that influence adopting the metaphors for new designs and example designs that the students created using the metaphors.

#### Users’ Perspective on the Use of Conceptual Metaphors: A HCI Course

In order to study users’ mental model on the use of three conceptual metaphors, we created and conducted a class activity, the Design by Metaphor learning activity, for a HCI course that introduces and provides experience in concepts and methods for HCI. The course is an active learning course that encourages learning through class activities. We developed a class activity to explore Design by Metaphor in three courses: 2019 summer (15 students), 2019 fall for undergraduate students (88 students), and 2019 fall for graduate students (43 students). We introduced the role of metaphors in HCI and examples of metaphors in HCI before conducting the class activity. We also introduced the three conceptual metaphors with example designs and design elements associated with each metaphor. The class activity is to identify signifiers, affordances, and interaction modalities in a given interactive design and describe which metaphor could be used to describe the design. For the activity, the students were given the three metaphors (device, robot, friend) and 18 examples of HCI designs in a template file including the definition of the three conceptual metaphors and a picture of each of the 18 example interaction designs. The 18 example designs for the activity include various types of interface and interaction (i.e. GUI, TUI, embodied, mobile, touch, mid-air gesture, speech, AR, VR, wearable, robotic, AI assistant). We selected the examples to represent different types of interaction, interface, modality, material, and appearance of design elements.

In the activity submissions, the students showed distinct patterns for mapping between the metaphors and design examples. The device metaphor was predominantly mapped to examples of GUI, mid-air gesture, and TUI. The robot metaphor was predominantly mapped to examples of physical movements of an artifact responding to users’ movements (e.g. a kinetic facade, an interactive wall, a travel suitcase) and a car infotainment. The friend metaphor was predominantly mapped to various AI assistants (e.g. Amazon Echo, Pillo, Gatebox). These patterns are consistent with the metaphorical framework that we identified from the review of existing embodied interaction designs shown in Table 1. In other words, the device metaphor is strongly associated with direct manipulation, the robot metaphor involves automated features and physical movements, and the friend metaphor reflects conversational interaction and a human-like manner.

Another interesting pattern is that some design examples were mapped to multiple metaphors. In these cases, the design examples mostly use multimodal interaction. For example, most students mapped either the robot metaphor or the friend metaphor to a personal assistant using GUI, touch, and speech interaction (e.g. Google Assistant app). Some students selected both the robot and the friend metaphor when mapping specific design factors to each metaphor. That means specific design factors such as the shape of the physical/digital design and the interface type affect selecting the metaphors.

We also identified the themes that the students described in mapping a design example to one of the metaphors. We identified three emerging themes from the answers to the question: *why did you select a specific metaphor for the signifiers, affordances, and modalities you found in the design example?* The three emerging themes are: signifier and affordance, interaction modality, and purpose of design. The students mostly focused on signifier and affordance and interaction modality in the device metaphor. That means when the metaphor is device, they focused on what the interface looks like and how it is controlled as a device. In the robot metaphor, the students mostly focused on interaction modality. In other words, they focused on the sensing type of interaction with automated features and physical movements of the robot metaphor. For the friend metaphor, the students focused on Purpose of Design associated with the concept of human-like manner and Interaction Modality associated with conversational interaction.

#### Designers’ Perspective on the Use of Conceptual Metaphors: An Interaction Design Studio Course

In order to study how designers apply the three metaphors to develop their conceptual design, we used the three metaphors in an interaction design studio course. The course is a studio approach to teaching topics in interaction design. Aspects of interaction design taught in the studio include: gesture-based interaction, tangible interaction, large public display interaction, tabletop interaction, multi-touch tablet interaction, and human–robot interaction. The topic of the design studio for the semester was metaphorical design for human–building interaction, and the design project focused on designing interactive buildings. The design project was a team project, and each team consisted of three to four students. During the semester, the students developed their design concepts based on the conceptual metaphors, implemented the prototype, and evaluated the prototype. We introduced metaphorical design and examples of metaphors in HCI at the beginning of the semester. We also introduced the three metaphorical concepts for HBI with examples, and the students performed precedent studies associated with each metaphorical concept during the first 3 weeks. After that, we assigned one of the metaphors (device, robot, or friend) that the students were asked to use when developing their design in their team project. There were six design teams, and we assigned each metaphor to two teams: two teams for the device metaphor, two teams for the robot metaphor, and two teams for the friend metaphor. The students used the assigned metaphorical concept and interaction type, interface type, and affordance in the design process as a framework for framing their conceptual design. In this section, we describe three design concepts that the students created and how the students applied each metaphor to develop their design concept.

The design concept influenced by the device metaphor is a smart door for a professor’s office that responds to visitors when the professor is absent. This design concept expanded the concept of an answering machine to an office door as a device. To be specific, when a student knocks on the door, the door greets the student and informs him/her that the professor is out for the day by playing a recorded voice, followed by prompting the student to leave a message with the student’s contact information. Once the student leaves a message, the door system immediately sends the student’s message to the professor by mobile app. The HCI techniques used for this design concept are:

• Interaction type: instructing
• Interface type: physical object, mobile
• Affordance/signifier: knocking to activate the system, “beep” sound for speaking, and LED lights for indicating the system status

This design concept presents a good example of metaphorical design in terms of mapping an existing concept of a device to a building component through analogical reasoning. The design team identified a novel function from an answering machine, and the answering machine was transferred to an office building. The design concept also shows a novel behavior for potential smart environments through the affordances and signifiers. The affordances and signifiers are a combination of physical and digital affordances that represent the characteristics of a door and an answering machine. As a result, designing the interaction model was strongly influenced by the metaphorical reference.

The design concept influenced by the robot metaphor is an interactive door for a classroom that provides class information. The interactive door detects students and displays the class information (e.g. class name, class schedule, number of students in the classroom) as a student approaches the door through emojis and messages. The emoji is a major design factor that indicates the class status. For example, when a student arrives at the door, the student can see a lit-up emoji with a neutral face and a small message “5 min left until the class database 101.” The HCI techniques used for this design concept are:

• Interaction type: sensing, exploring
• Interface type: display
• Affordance/signifier: emojis for indicating the class status

The design team focused on two aspects to adopt the robot metaphor: the automated sensing and the visual interface for the system. The automated features in the design are detecting users, collecting spatial data, and displaying the building information to increase the users’ awareness on their activities and the building status. While the automated features represent the characteristics of the robot metaphor, the emoji visualization makes the design look more like a typical robot.

The design concept influenced by the friend metaphor is a smart speaker, an office assistant called Pluto, for a meeting room. The smart speaker, which has a 3D-printed alien shape, is a voice-activated recording system for a meeting room. It detects the users’ presence in the room and responds to the users’ speech and records a group conversation or individual presentation, and then it exports a text transcript to an email or a Google Drive folder. The used HCI techniques for this design concept are:

• Interaction type: conversing
• Interface type: speech, appliance
• Affordance/signifier: greeting the users for activating the system, speaking instructions for recording the conversation or sending the file, LED lights on the ears of the 3D-printed alien for indicating the recording status

The design concept is similar to existing smart speakers such as Amazon Echo. However, this design shows an example of an embedded AI assistant in a built environment focusing on the meeting activities and the meeting space. That means the smart speaker has a clearer role as an office assistant for the meeting room, while existing smart speakers play more universal roles, mostly focusing on the smart home. Another aspect in adopting the friend metaphor in this design concept is the shape of the smart speaker. The smart speaker was designed as a 3D-printed alien shape, and the ears of the alien include LED lights to indicate that the alien is hearing the users’ voice. As a result, the combination of speech interaction, the physical shape of the design, and the signifier make the design more like a friend through personification of the interactive system.

### Framing a Single Product That Is Developed for Each of the Metaphorical Concepts

The three metaphorical concepts we present provide distinct design spaces based on the characteristics of each metaphorical concept and the HCI techniques associated with each metaphorical concept. The metaphorical concepts provide a conceptual framing for a design, and the HCI techniques provide a technical framing that facilitates the exploration of the design space in a practical way. Each metaphorical concept thus can guide a design to different concepts for the same purpose or the same function through the metaphorical framework. In this section, we describe how a specific design for smart environments can be conceptualized differently by the different metaphorical concepts.

The smart home and energy conservation are common examples for smart environments. Many smart home apps and smart devices provide similar functions for energy saving such as managing room temperature, but they use different interactions and interfaces. We describe how each metaphorical concept can frame designing a smart room for energy conservation as an example. We apply the same design goals and functions to the three metaphorical designs for the smart room design. The design goals are saving energy and satisfying environmental comfort. The functions are managing energy consumption, controlling room temperature, controlling lighting, and controlling shading.

Smart room as device. The device metaphor for smart environments provides the conceptual space for easy control in the built environment. In order to achieve the design goals and the functions, this conceptual space specifies the instruction type of interaction and includes possible interface types focusing on how to realize easy control. To be specific, information visualization, sharable, display, and mobile are possible interface types to achieve the function of managing energy consumption. For the function of controlling room temperature, lighting, and shading, GUI, TUI, touch, mid-air gesture, and mobile are possible interface types. While interface types frame a technical solution, affordances and signifiers guide detailed designs. In the device metaphor, metaphorical references provide visual and physical cues for affordances and signifiers, and the use of metaphorical references makes the design more like a device supporting a user’s mental model. For instance, a dashboard design, icons, switches, dials, buttons, and sliders can be applied to the information visualization for indicating the energy consumption as a digital affordance. Vibrating blinds, flickering bulbs, beep sounds, and different colors of LED indicator on the wall can indicate a certain energy status and afford a user action to save energy as a physical affordance. The affordances associated with the device metaphor also help to design user actions (e.g. gesturing up/down, a dial shape of TUI, and tapping the wall) for controlling room temperature, lighting, and shading. These digital/physical affordances based on the interface types help to achieve the design goal of energy saving supporting the function of managing energy consumption and the design goal of satisfying environmental comfort supporting direct manipulation based on the user’s preference.

Smart room as robot. The robot metaphor for smart environments captures the design space for autonomous features of smart environments. This conceptual space can expand the given functions, managing energy consumption and controlling room temperature/lighting/shading to collecting environmental data, tracking energy consumption history, and analyzing the user’s pattern. These expanded functions then are transferred to specific system actions based on the sensing and exploring types of interaction. The expected system actions enable a personalized setting for providing comfort and optimizing environmental conditions for saving energy such as automatic changes of blinds and temperature. The physical system actions make the smart room a robotic building component. In this case, the design does not emphasize affordances since it does not require intentional user actions. For the metaphorical design using the robot metaphor, the focus is on the automated features and identifying novel functions/behaviors that can be automated by the system using additional sensors.

Smart room as friend. The friend metaphor provides the design space for personified smart environments that support occupants’ personal activities. This metaphorical reasoning can be applied to a smart room (e.g. personified room) or a building component (e.g. personified door) through the use of a virtual character. The interaction types associated with the friend metaphor are conversing and exploring. In this case, the user saves energy and controls the environment through conversational interaction with the personified room. For example, a virtual character recommends opening/closing window blinds based on changes of external conditions, and the user confirms the change. Another example is that the virtual character gives energy saving tips based on the user’s behavioral patterns. In this case, the smart room encourages a user’s behavioral changes in the use of energy and increases the user’s awareness. Selecting interface types for the friend metaphor depends on the visibility of the virtual character. Speech and/or display for chatting can be considered for an invisible virtual character, and AR and speech can be possible interfaces for a visible virtual character. For the personification of the environment, the smart room design can use different metaphorical references for the role of the friend (e.g. energy expert, building manager, buddy, colleague, and roommate). The role of the friend influences the design of the virtual character and the signifiers for the affordances (e.g. speaking manner, gestures, and actions).

## Conclusion

Metaphors provide a common perspective for characterizing new designs in HCI. Metaphors assist in forming a common mental model for new interactive designs that support designers in creating novel interaction experiences. Embodied interaction is an integral aspect of smart environments due to the scale of the environment. As recent embodied interactions increasingly expand to include new technologies with multimodal interaction, smart environments provide potential design spaces that are yet to be fully explored and understood. Characterizing smart environments with embodied interaction by using conceptual metaphors can be a new foundation for a theoretical and methodological framework to understand and discover the potential design spaces for smart environments. In this paper, we focus on how people interact with the built environment, while many architects focus on how advances in technology can improve the quality of the built environment using automation.

We present three metaphorical concepts that enable new ways of designing smart environments: device, robot, and friend. The device metaphor represents performing tasks through the users’ direct control; the robot metaphor emphasizes automation, autonomous decision making, and the physical actions of a robot; and the friend metaphor represents performing tasks using human-like assistance for supporting users’ activities.

A critical review of existing embodied interaction designs using the three metaphorical concepts with HCI techniques provides a framework for characterizing design spaces for smart environments. We reviewed 24 existing embodied interaction designs that represent various interaction modalities for embodied interaction. The analysis shows that each metaphorical concept refers to distinct interaction types, interface types, and affordances, which creates distinct design spaces for smart environments. We found that the device metaphor is associated with instructing, manipulating, and exploring types of interaction. These interaction types involve TUI, mid-air gesture, wearable, and OUI as interface types. The affordances and signifiers for the device metaphor use generalized GUIs and physical shapes of an interface to make the designs look like a physical device.

While the device metaphor focuses on direct manipulation, the robot metaphor focuses on automated features using exploring and sensing types of interaction. The interface types associated with the robot metaphor are robot, appliance, ambient device, and OUI with automation. Since these interface types rely on a sensor-based system in a physical space, the affordances and signifiers for the robot metaphor involve physical movements of occupants and/or architectural components rather than graphics and physical shapes.

The friend metaphor reflects conversational interaction and a human-like manner based on the personification of the interactive system, while the device and the robot metaphor reflect functional aspects of the artifact. The interaction types for the friend metaphor thus include conversing and exploring. The primary interface type for the conversing type of interaction is speech, but it can be expanded to many other interface modalities with dialog features and applications such as appliances and wearable devices. The affordances and signifiers for the friend metaphor are unique and interesting in the sense that it uses speaking instruction, emotional speech, tone, pace, pitch of speech and actions of a virtual assistant associated with physical or graphical shapes. Identifying new affordances and signifiers for the friend metaphor is a topic for research on novel interaction designs for smart environments.

The educational experiences in which we presented the three metaphorical concepts show the effect of the conceptual metaphors on recognizing affordances in smart environments and on designing new smart environments. We focused on how people adopt the metaphors as a mental model from the user’s perspective and how designers apply the three metaphors to develop their conceptual design from the designer’s perspective. From the user’s perspective, the students showed distinct patterns on mapping the metaphors to design examples that are consistent with the metaphorical framework identified from the review of existing embodied interaction designs. That means the metaphorical concepts can provide a sharable mental model for new designs. We also identified the influential factors in adopting each metaphor: signifier and affordance and interaction modality in the device metaphor, interaction modality in the robot metaphor, and purpose of design and interaction modality in the friend metaphor. These findings support the effect of the metaphorical design framework on users’ perspective, but further research is needed to collect statistically significant data in a laboratory study in order to verify the effect of the metaphorical framework. From the designer’s perspective, the conceptual designs that the students created show how designers use the metaphorical framework to develop a design concept for smart environments. The design applying the device metaphor actively used a metaphorical reference (i.e. answering machine), a building component (i.e. door), and physical affordance (i.e. knocking). The design applying the robot metaphor focused on automated features and the visual representation of the system. The design applying the friend metaphor showed a unique role of a friend in a specific environment (i.e. office assistant), an appearance of the personified object (i.e. alien), and the signifiers for the affordances of a virtual assistant (i.e. LED indicator on the ears and the speaking manner). These results show different aspects of metaphorical design when applying the metaphors and framework for a specific design. The limitation of these results is in the scale (20 participants) and context (design education) of the study. Further research is needed to conduct a laboratory study of the three metaphors that recruits more participants and studies the effect across several design tasks.

We explored how each metaphorical concept can conceptualize a specific design differently through framing a smart room design for energy conservation. The exploration of a smart room design using the three metaphorical concepts shows that each metaphorical concept facilitates: achieving the design goals by providing a distinct conceptual space, selecting interaction types based on each metaphorical concept as a guide to explore possible interface types, and guiding the designer toward specific affordances and signifiers based on metaphorical references when designing the interactions, user actions, and interfaces. In framing the smart room design, the device metaphor provided the conceptual space for easy control to achieve the design goals, and the interaction type and the interface type focused on how to realize easy control. Physical affordances in the device metaphor involved physical building components to make the smart room a device, and the combination of digital and physical affordances influenced the design of user actions using the metaphorical references. While the conceptual design in the device metaphor actively used metaphorical references for designing user actions, the conceptual design in the robot metaphor focused on identifying system actions associated with the automated features. The conceptual space for autonomous features facilitated expanding and transferring the given functions to automated functions, for example, controlling the room temperature by tracking/analyzing the occupants’ patterns of behavior in the room. This metaphorical design thus provides a basis for identifying novel automated functions and behaviors of the system. The friend metaphor provided the design space for personified smart environments. In the metaphorical design using the friend metaphor, the role of the friend provided a basis for considering the interaction types and interface types. The metaphorical references for the role of the friend influenced the design of the affordances, and this metaphorical design facilitated identifying novel personalized and friendly functions and behaviors for the interaction.

We conclude that the metaphorical concepts presented can frame new design spaces that lead to a shared mental model and novel interaction designs for future smart environments. The metaphorical concepts can be a design tool and an educational tool for designing smart environments. The contribution of this paper is a review of existing embodied interaction designs from the perspective of three metaphorical concepts and a metaphorical design framework that enables novel approaches to conceptualizing interactivity in smart environments as a device, robot, or friend.

## Author Contributions

JK composed this study, designed the framework, and completed the analysis. MM provided supervision throughout the research and contributed substantially to the analytical part of the research.

## Conflict of Interest

The authors declare that the research was conducted in the absence of any commercial or financial relationships that could be construed as a potential conflict of interest.

## Footnotes

1 Amazon Echo (2014). *— Alexa Speaker*. Available online at: https://www.amazon.com/all-new-amazon-echo-speaker-with-wifi-alexa-dark-charcoal/dp/B06XCM9LJ4 (accessed February 28, 2020).

2 Goji Smart Lock (2014) *Indiegogo*. Available at: http://www.indiegogo.com/projects/400022/fblk (accessed February 28, 2020).

3 Travelmate Robotics (2018). *Travelmate Robotics*. Available at: https://travelmaterobotics.com/ (accessed February 28, 2020).

4 Gatebox (2019) *Gatebox*.  Available at: https://www.gatebox.ai (accessed February 28, 2020).
